# Enhancement in sustained friction and wear resistance of nanoporous aluminum oxide films obtained by controlled electrochemical oxidation process

**DOI:** 10.1039/c9ra05702a

**Published:** 2019-08-12

**Authors:** Lidia Benea, Valentin Dumitrascu

**Affiliations:** Competences Center: Interfaces-Tribocorrosion-Electrochemical Systems (CC-ITES), Dunarea de Jos University of Galati 47 Domneasca Street RO-800008 Galati Romania Lidia.Benea@ugal.ro https://www.cc-ites.ugal.ro +40 744216277

## Abstract

The primary purpose of this study is to investigate the effect of the electrochemical parameters required in the anodic oxidation process on the friction and wear resistance of the obtained nanoporous aluminum oxide films. The wear resistance of the aluminum oxide films and the electrochemically polished Al1050 alloy were tested using a ball-on-disc tribometer having a corundum ball as counterbody in dry conditions. The friction coefficient graphs were recorded during wear tests at a 5 N normal force simulating the application of nanoporous aluminum oxide films in industrial areas requesting moderate wear resistance. The wear tracks formed on the tested surfaces were analyzed *ex situ* both qualitatively and quantitatively. Higher wear resistance is showed by nanoporous aluminum oxide films as compared with electro polished Al1050 alloy substrate.

## Abstract 2

The primary purpose of this study is to investigate the effect of the electrochemical parameters required in the anodic oxidation process on the friction and wear resistance of the obtained nanoporous aluminum oxide films.

## Graphical abstract

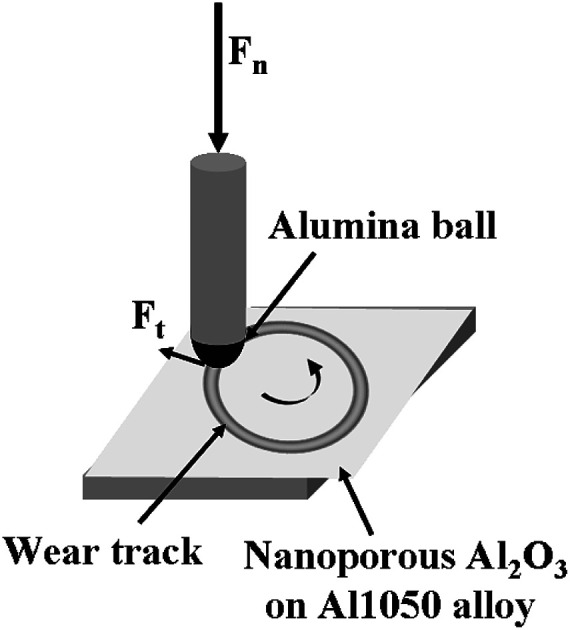

## Introduction

Aluminum and its alloys, due to their properties such as low density, high strength and non-magnetic characteristics, electrical and thermal conductivity, have become the second most used material in terms of applicability.^1^

Even if aluminum and its alloys, after exposure to oxygen atmosphere, are covered by a thin native aluminum oxide film, the anticorrosive and tribological properties are poor when aluminum and its alloys are exposed to an aggressive environment.^1^

In order to improve the anticorrosive and tribological properties of aluminum and its alloys, different surface treatment methods are used.^2–4^

Anodic oxidation process or electrochemical oxidation is a traditional surface treatment technology used to improve their surface properties.^5^

The anodic oxidation process converts the aluminum to aluminum oxide (Al_2_O_3_) by applying an external potential/current in the presence of an electrolyte.^6,7^

The anodic oxide film thus obtained features excellent anticorrosive performance, improved paint adhesion and good tribological properties.^3,8–10^

The requirements of lightweight materials for manufacturing different components for automotive (fuel handling tank systems for race cars running on alcohol, methanol, ethanol, gasoline, diesel fuel, high pressure fittings, for hydraulic lines, brake lines, cooling lines, and brake calipers, motorcycle front forks) and aerospace industries (helicopter blades, exterior panels for aerospace vehicles) need more investigations from the tribological point of view. Despite several studies on tribological performance, the dry sliding wear behaviors of nanoporous aluminum oxide film in the absence of solid lubricants need to be further investigated.

The novelty of this research work consists in the effect of the electrochemical parameters imposed in the anodic oxidation process on the wear resistance (mechanical properties) of the obtained nanoporous aluminum oxide films. The wear resistance of the aluminum oxide films and the electrochemical polished Al1050 alloy were tested using the ball-on-disc tribometer.

The friction coefficient graphs recorded during wear tests at a 5 N normal force simulating the application of nanoporous aluminum oxide films in industrial areas, where the materials used must exhibit cumulative properties like: low weight, weldability, corrosion resistance and/or moderate wear resistance, *etc.* The wear tracks formed on the tested surfaces of nanoporous aluminum oxide films were analyzed *ex situ* both qualitatively (Scanning Electron Microscopy) and quantitatively (volume loss and wear rate).

## Experimental

### Fabrication of nanoporous aluminum oxide films

In this investigation a 2 mm thickness 1050 aluminum alloy (Al1050, 99.5% Al) plate was used as substrate and cut into samples of 35 × 30 mm dimensions. The Al1050 samples were mechanically wet-polished with grinding paper (#1500 and #2000) until all scratches were no longer visible and chemically etched in 5 M sodium hydroxide for 30 seconds before use. The Al1050 samples prepared as shown above were electropolished in 15% sodium carbonate and 5% sodium phosphate at 80 °C and 2 V applied potential for 30 minutes,^11^ cleaned with distilled water and dried under heated air flow.

The anodizing processes were performed in a standard 2 electrode cell, containing 1 M sulfuric acid in which 1 g L^−1^ aluminum sulfate octadecahydrate was added. An electric DC power source (TDK LAMBDA GEN 300-8) was used to control the applied potential during the anodizing of electropolished Al1050 samples against a large untreated Al1050 plate as counter electrode.

The anodic oxidation process was performed for 45 minutes at room temperature for different applied potentials and the static electrolyte. After the anodic oxidation process, the obtained nanoporous aluminum oxide films were cleaned using distilled water, dried at 50 °C for 60 minutes and prepared for future characterization.

## Results and discussions

### Characterization of nanoporous aluminum oxide films

The surface morphology and thickness of nanoporous aluminum oxide films were examined using scanning electron microscope (SEM) Fei Quanta 200 coupled with an X-ray energy dispersive (EDS) analyzer before the friction and wear tests.

The friction and wear tests were conducted in non-lubricated conditions, ambient air and a pin-on-disk configuration, using the TRM1000 (Wazau, Germany) tribometer. A commercially available grade 10 corundum ball (Ceratec, Netherlands) with a diameter of 10 mm was used as counter body. The corundum ball was selected as a counterbody due to its high wear resistance, high chemical inertness and high electrical resistance. Before the friction and wear testing, the corundum balls were cleaned with alcohol.

**Fig. 1:**
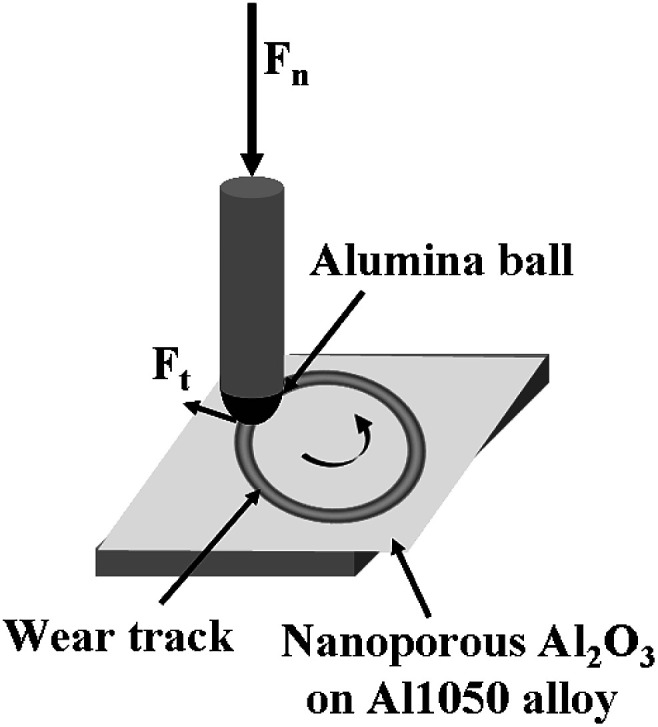
Schematic drawing of the contact between the electrochemically polished or oxidized aluminum alloy surface and the alumina ball during tribological tests.

The schematic drawing of wear test setup is shown in Fig. 1.

The friction tests were conducted by applying an external force of 5 N, the rotating velocity was 0.01 m s^−1^ for 50 minutes resulting a wear track with 10 mm diameter. The wear tests of the nanoporous of aluminum oxide films used a normal 5 N force for 50 minutes and at a rotational speed of 9.55 rpm. During the friction test, the normal force, tangential force and coefficient of friction were continuously monitored. After the friction tests, the samples were ultrasonically cleaned successively in alcohol and distilled water for 5 min and dried in warm air.

The wear track surface morphologies were investigated using the scanning electron microscope and the cross-section profiles of worn surfaces were measured using a 2D profilometer (Mytutoyo Surftest SJ-210, Japan). The 3D profiles of wear tracks were obtained using the image analysis software ImageJ (http://www.imagej.nih.gov/ij), (National Institute of Health, USA) that was used to acquire qualitative information on the wear tracks from the SEM micrographs recorded. The SEM micrographs were at first transformed into a binary format whereupon a transformation function was applied to project the gray intensity to *Z* direction. The wear rate (*k*) was calculated using the following formula (1):1
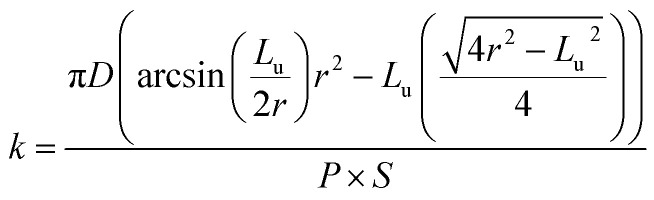
where: *r* is the radius of corundum ball (mm), *D* is the diameter of wear track (mm), *L*_u_ is the width of wear track (mm), *S* is sliding distance (m) and *P* is applied normal load (N).

The wear rate (*k*) was calculated using the eqn (1). The wear track volume (*V*_u_) was calculated using the eqn (2).2
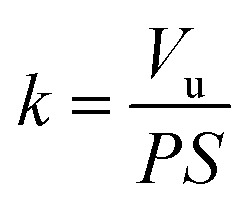
where: *V*_u_ is the wear volume in mm^3^; *P* is the normal force (5 N) and *S* is sliding distance (30 m).

The wear loss volume and wear rate graphs were recorded after these calculations.

### Fabrication and characterization of nanoporous aluminum oxide films

Before the anodic oxidation process, the 1050 aluminum alloy samples were degreased, etched and electropolished, in order to obtain smooth surfaces with no visible defects.

**Fig. 2:**
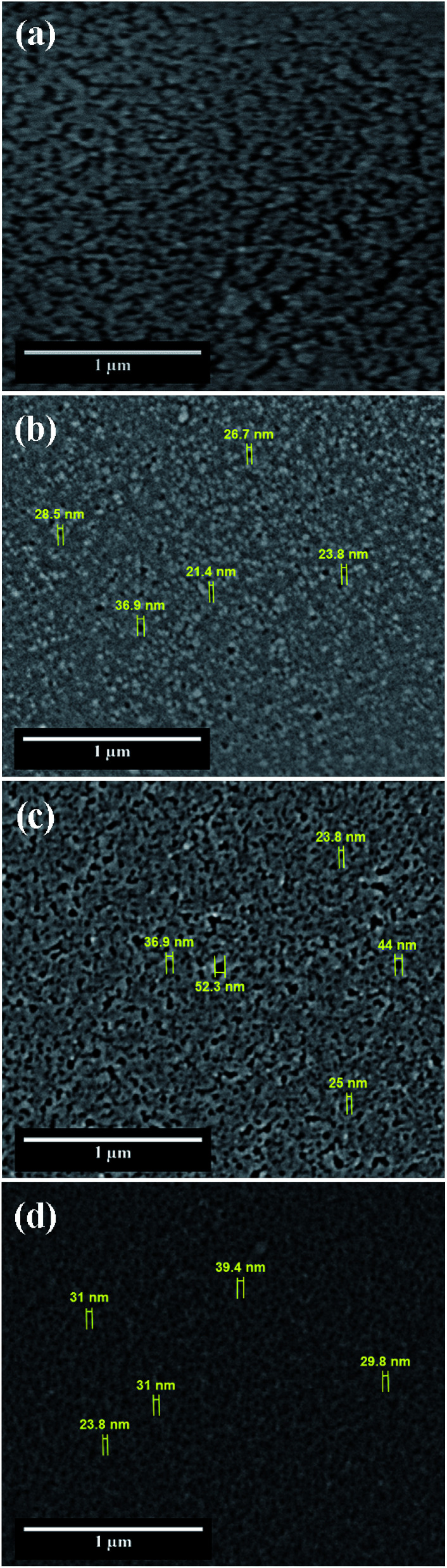
SEM surface morphology of: (a) electrochemically polished Al1050 alloy; (b) nanoporous Al_2_O_3_ film formed at 15 V imposed potential; (c) nanoporous Al_2_O_3_ film formed at 18 V imposed potential and (d) nanoporous Al_2_O_3_ film formed at 21 V imposed.

The SEM image of electropolished Al1050 surface is presented in Fig. 2a at a higher magnification and show a non-uniform surface composed by randomly located depressions, channels and nanopits that act as nucleation centers for nanopores grown during the further anodic oxidation process.

After the electrochemical polishing treatment, the Al1050 samples were anodized, in one-step process, in 1 M H_2_SO_4_ in which was added 1 g L^−1^ Al_2_(SO_4_)_3_·18H_2_O at different voltages.

During the anodic oxidation process, the anodic oxide films grow at the anode electrode according to the reactions (3) and (4):^12^

3 6OH^−^ − 6e^−^ → 3H_2_O + 3O^2−^

4 2Al + 3O^2−^ → Al_2_O_3_(amorph)

At the cathode, the hydrogen evolves as follows (eqn (5)):

5 6H^+^ + 6e^−^ → 3H_2_(gas)

Simultaneously phenomena occur during the anodic oxidation process: the native aluminum oxide film is dissolved under the aggressive action of electrolyte and electric field, followed by alumina barrier growth and formation of porous oxide film driven by the ionic diffusion of O^2−^/Al^3+^ ions across the barrier film under the effect of electric field and concomitant dissolution of formed alumina film (porosity increasing).^13^ The morphologies of fabricated nanoporous aluminum oxide films on Al1050 surface for 45 minutes at 15 V, 18 V and 21 V and a static electrolyte regime are shown in Fig. 2b–d.

The nanoporous aluminum oxide films are clearly seen and obtained on all 3 applied potentials. The morphology of Al1050 sample anodized at 15 V Fig. 2b reveals randomly distributed nanopores with non-uniform diameters and interpores distances.

Increasing the anodizing potential from 15 V to 18 V, the SEM micrograph Fig. 2c also increases the nanopores density but maintains a random distribution, irregular diameters and non-uniform interpores distances. For the nanoporous aluminum oxide film obtained at 21 V Fig. 2d, the nanopores density increases even more as compared with the nanoporous aluminum oxide films obtained at 15 V and 18 V, respectively. The nanopores distribution is almost ordered, the nanopores diameters and interpores distances are nearly uniform distributed over the analyzed surface.

**Fig. 3:**
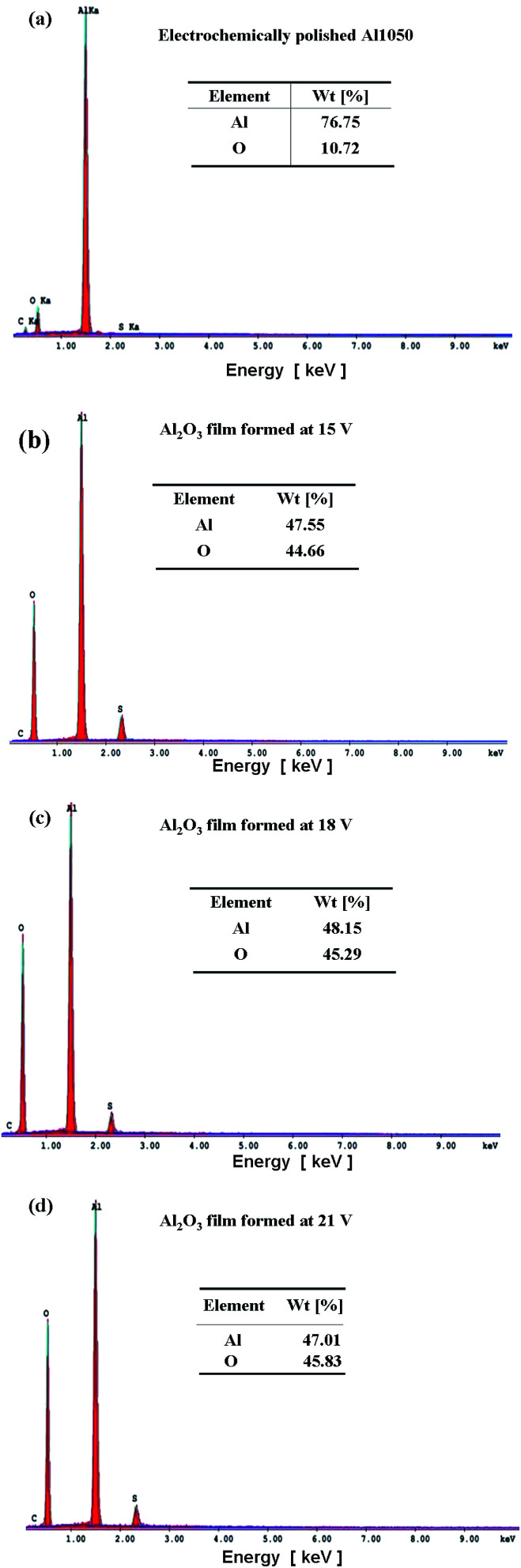
SEM-EDX surface spectrum analysis of: (a) electrochemically polished Al1050 alloy; (b) nanoporous Al_2_O_3_ film formed at 15 V imposed potential; (c) nanoporous Al_2_O_3_ film formed at 18 V imposed potential; (d) nanoporous Al_2_O_3_ film formed at 21 V imposed potential.

Fig. 3 shows the EDX spectrum of electropolished Al1050 Fig. 3a, anodized Al1050 at 15 V for 45 minutes and a static electrolyte regime Fig. 3b, at 18 V for 45 minutes and a static electrolyte regime Fig. 3c and 21 V for 45 minutes and a static electrolyte regime Fig. 3d. The figure shows that the anodizing process increases the weight percentages corresponding to oxygen from 10.72% to 45.83%. The same tendency of increasing the oxygen percentage simultaneously with the increasing of applied anodic potential has been reported by other authors on oxidation of pure aluminum^14^ and Al1092 alloy.^15^

### Thickness of nanoporous aluminum oxide films

**Fig. 4:**
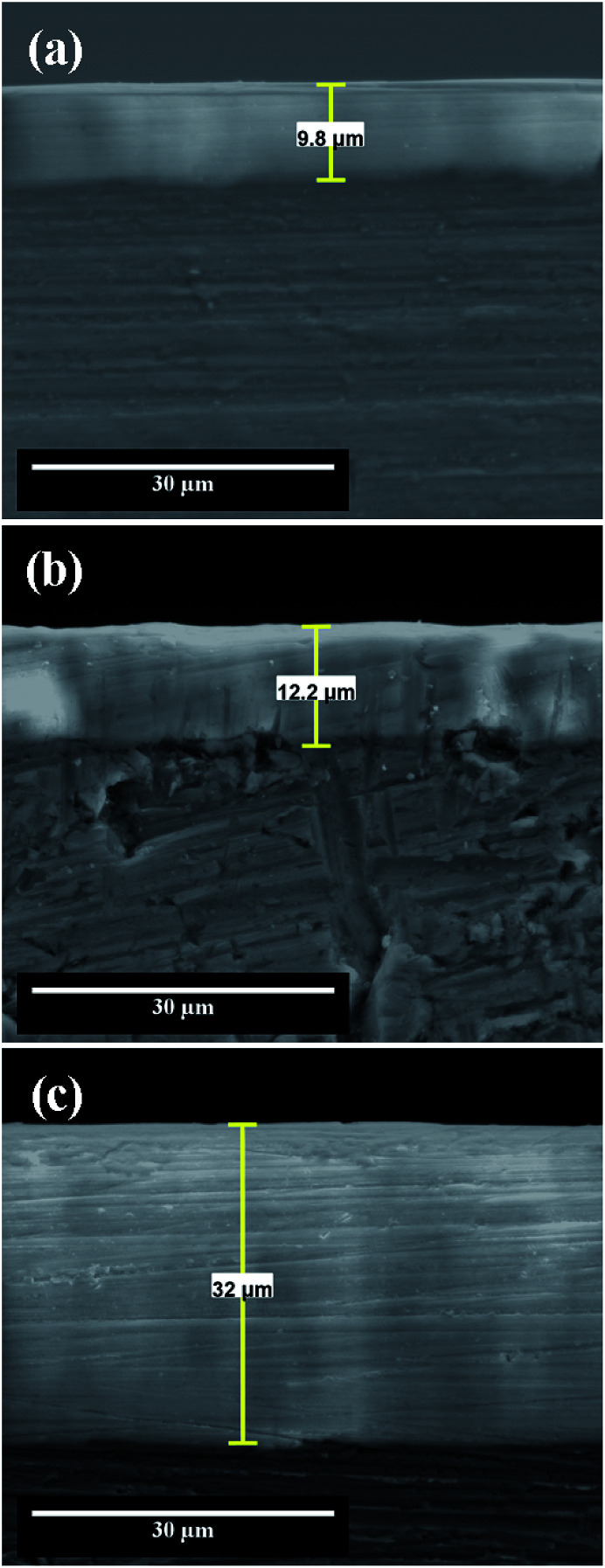
SEM cross section micrographs of nanoporous aluminum oxide films obtained by electrochemical oxidation during 45 minutes at: (a) 15 V; (b) 18 V; (c) 21 V.

The SEM cross-section view of nanoporous aluminum oxide films obtained at different potentials in static electrolyte conditions reveal that the anodic films thickness increases simultaneously with the applied potential from 9.8 μm to 32 μm, as shown in Fig. 4. From SEM cross-section morphologies it cannot be identified fractures at the Al1050 substrate-oxide film interface, revealing that the anodic oxidation films are well bonded with the Al1050 substrate. Even if, from the top-view SEM micrographs, the anodized films present a porous structure that cannot be seen in the cross-section SEM micrographs, this was destroyed during wet-polishing with hard SiC abrasive paper, used to prepare the cross-section of samples for SEM investigations, as pointed out also by other authors as well which investigated the Al6061 alloy anodized in sulphuric acid 20% with applied potential of 25 V to 70 V.^16^

**Fig. 5:**
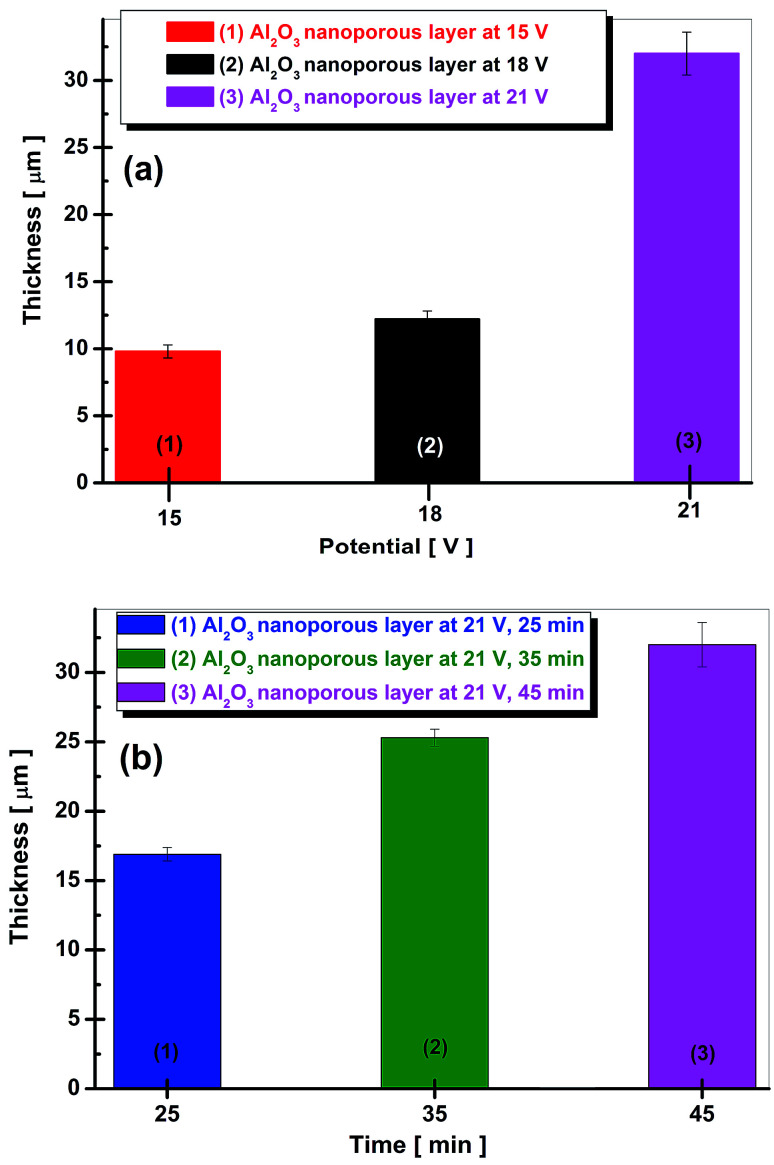
Evolution of film thickness: (a) *vs.* applied potential to obtain nanoporous aluminum oxide film; (b) *vs.* duration of anodic oxidation process to obtain nanoporous aluminum oxide film.

The thickness of anodic oxide films depends on the applied anodic potential as plotted in Fig. 5a and on the duration of anodic oxidation process as plotted in Fig. 5b.

The thickness of nanoporous aluminum oxide films increases simultaneously with the applied potential during electrochemical oxidation process as well as with time of oxidation process at the constant applied potential.

### Wear performances of nanoporous aluminum oxide films

#### Friction coefficient

The coefficient of friction is an important factor determining the choice of material for use in specific environments.

Even if friction coefficient values are standardized, in practice working and environmental conditions affect them.

In order to determine the actual values of the friction coefficients corresponding to the materials used in specific environments, the laboratory test conditions must be the same as those used to identify the parameter or set of parameters that determine their change.

**Fig. 6:**
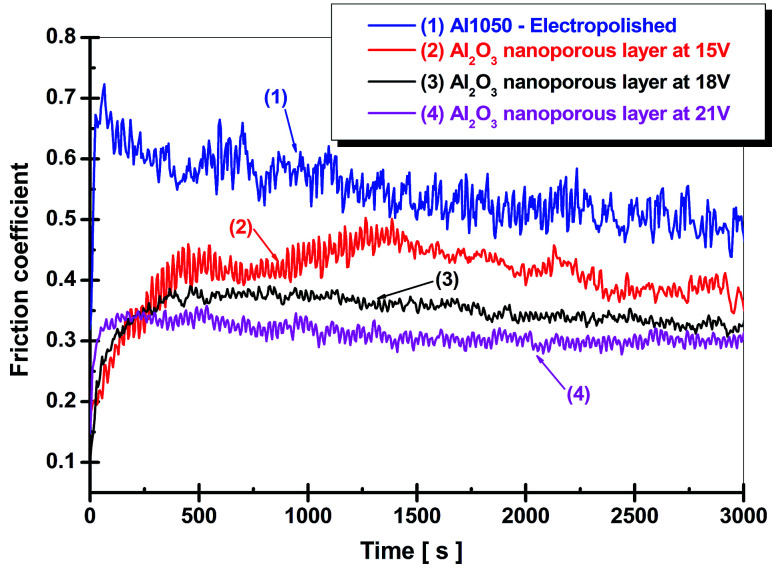
Friction coefficients of electropolished aluminum alloy and nanoporous aluminum oxide films *versus* time. Influence of the anodic oxidation potential on the variation of the friction coefficients: (1) electrochemically polished Al1050 alloy; (2) nanoporous Al_2_O_3_ film formed at 15 V imposed potential; (3) nanoporous Al_2_O_3_ film formed at 18 V imposed potential and (4) nanoporous Al_2_O_3_ film formed at 21 V imposed potential.

Fig. 6, shows the friction coefficient diagrams for electrochemically polished aluminum alloy and nanoporous aluminum oxide films obtained by anodic oxidation during 45 minute of electrochemical oxidation at the imposed potentials of 15 V, 18 V and 21 V.

From Fig. 6 it can be seen that the friction coefficient diagram corresponding to the electrochemically polished aluminum sample Al1050 has a decreasing trend with the increasing of the sliding distance or with time due to the formation of a tribo-layer in the contact area between the sample and the pin (alumina ball, corundum). The average value recoded for friction coefficient of only electropolished aluminum alloy was 0.546. Fig. 6 that the friction coefficients recorded for the nanoporous aluminum oxide films obtained in a static electrolyte during the anodic oxidation process exhibit lower average values by increasing the imposed potential in the anodic oxidation process.

The same trend of decreasing the coefficient of friction for the nanoporous film of aluminum oxide compared to the friction coefficient of the pure aluminum substrate was observed by H. Wang and his team.^17^ A decrease in the friction coefficient corresponding to the nanoporous titanium oxide film growth by anodic oxidation on the Ti–6Al–4V titanium alloy surface compared to the friction coefficient corresponding to the Ti–6Al–4V titanium alloy mirror polished was reported by some authors.^18^ For the aluminum oxide film formed at a potential of 15 V, the highest average value of friction coefficient was recorded and found at a value of 0.407. Also, from the diagram presented in Fig. 6, there are large fluctuations in the coefficient of friction, implying degradation of the aluminum oxide film. For nanoporous alumina films formed at 18 V and 21 V, the coefficient of friction shows average values of 0.348 and 0.311 respectively being lower as compared to the average friction coefficient recorded for oxide film obtained at 15 V. Nanoporous aluminum oxide films obtained by anodic oxidation show a decrease in the average values of the friction coefficients with the increase of the imposed potential during electrochemical forming process which results in increased thickness and implicitly improved mechanical properties.

#### Wear track surface analysis

**Fig. 7:**
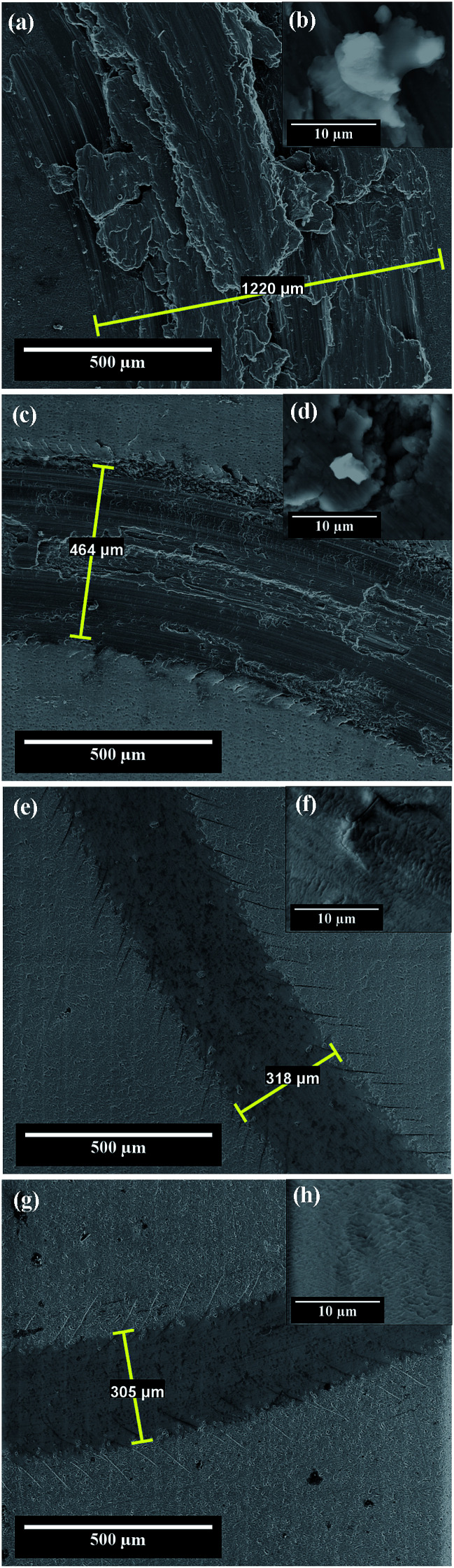
SEM micrographs corresponding to the wear pattern formed on: (a) the electrochemically polished Al1050 substrate; (c) nanoporous aluminum oxide film obtained at 15 V imposed anodization potential; (e) nanoporous aluminum oxide film obtained at 18 V imposed anodization potential; (g) nanoporous aluminum oxide film obtained at 21 V imposed anodization potential. (b, d, f and h) are SEM micrographs of wear debris inside the tracks at a higher resolution corresponding to the tracks (a, c, e and g).

The morphological characterization of the wear tracks was done using the scanning electron microscope. The wear track width was determined at four cardinal points on the surface of the wear track, and then the arithmetic mean value of the wear track was calculated. The SEM micrographs in which the wear track width value is the closest value to the mean value of the wear track are presented in Fig. 7.

Fig. 7a shows the wear track formed on the electrochemically polished surface of the Al1050 substrate subjected to mechanical tests applying a normal force of 5 N for 50 minutes and a rotational speed of 9.55 rpm. Fig. 7a shows that the width of the wear track has an average value of 1220 μm. In the contact area between the pin and the aluminum surface there is a formation of a tribo-layer having a discontinuous structure, with a large amount of wear debris, material ruptures and crevices visible as shown in Fig. 7b.

Fig. 7a and b shows that the wear debris has different dimensions and shapes. Due to the fact that both the aluminum and its alloys feature increased ductility, an adhesive wear is in progress/takes place on the surface. The wear residues formed during the wear tests are not completely removed from the surface of the aluminum sample and they adhere to the tribo-layer formed in the contact area between the sample and the pin during the friction process as illustrated in Fig. 7a and b.

Some authors^19^ also observed the phenomenon of adhesive wear on the surface of untreated Al1050 subjected to wear in dry condition at a normal force of 9 kN.

Fig. 7c and d shows the SEM micrographs of wear tracks formed on aluminum oxide nanoporous surfaces obtained over a period of 45 minutes in a static electrolyte regime and to an imposed potential of 15 V.

Compared to the wear track diameter shown in Fig. 7a, the wear track width formed on the surfaces of the nanoporous aluminum oxide films show much lower values as illustrated in Fig. 7c and d. The wear track width has an average value of 464 μm and the tribo-layer formed in the contact area between the pin and the oxide surface is not uniform, showing ruptures of material, uneven wear and cracks visible on its surface. For oxide layer obtained at 15 V, in Fig. 7c and d there are shown some wear debris appearing in the wear track and more clearly in Fig. 7d there are observed the penetration cracks into the oxide layer.

The SEM micrographs from Fig. 7e and f show the wear track formed on the surface of the nanoporous aluminum oxide film obtained at 18 V potential having an average diameter of approximately 318 μm of the wear track. The tribo-layer formed in the contact area between the pin and the oxide surface shows a much lower number of wear residues but at the same time cracks are visible both inside the wear track as shown in Fig. 7e as well as on the wear track borders, due to abrasive wear and stress accumulated in the contact area, as it was observed by other authors on Al1050 alloy anodized subjected to wear in dry condition at a normal force of 9 kN.^19^

Fig. 7g and h shows the SEM micrographs of the wear track formed on the surface of the nanoporous oxide film obtained by anodic oxidation at a potential of 21 V. The wear track average width value is 305 μm. The tribo-layer formed in the contact area between the aluminum oxide film and pin (alumina ball) exhibits a small number of cracks with a relatively low depth, compared to the tribo-layer formed on the surface of the aluminum oxide film obtained at a potential of 18 V. Also, at a higher magnification Fig. 7h the SEM micrograph recorded inside the wear track does not show any cracks or wear debris.

The only electrochemically polished aluminum alloy surface exhibited an adhesive wear while the nanoporous aluminum oxide films obtained by anodic oxidation on the surface of the 1051 aluminum alloy in a sulfuric acid electrolyte exhibited abrasive wear.

### Quantitative characterization of wear tracks. Wear volume and wear rate

In order to evaluate the tribological properties of the nanoporous aluminum oxide films obtained by anodic oxidation, wear tests determined the wear volume (the amount of material lost during the mechanical tests) as well as the wear rate.

**Fig. 8:**
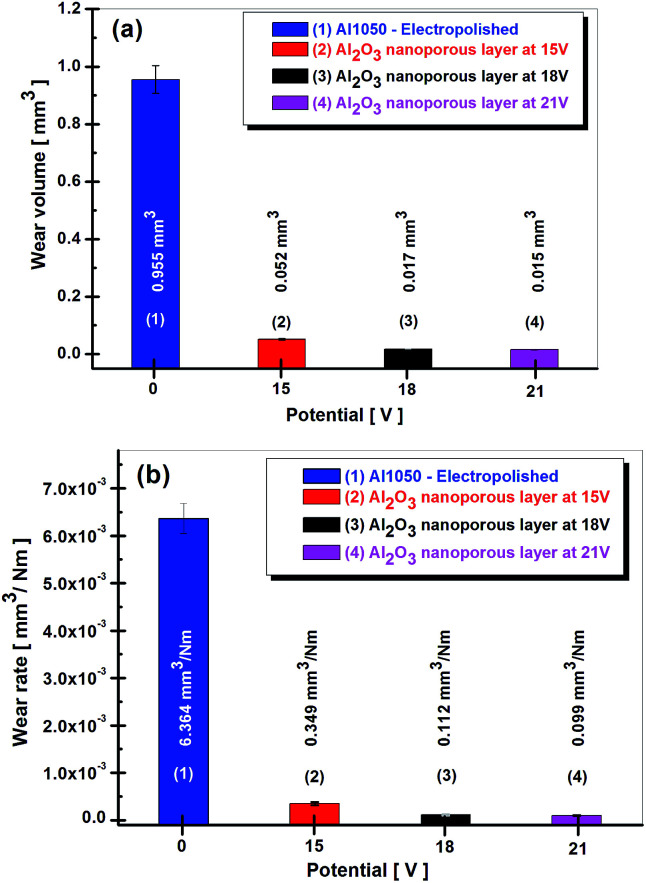
Wear volume loss calculated from wear tracks (a) and wear rate (b) for electro polished Al1050 alloy nanoporous aluminum oxide films obtained at imposed potential of 15, 18 and 21 V.

The wear volume and wear rate are shown in Fig. 8. From the values of the wear volume losses presented in Fig. 8a, there is shown a decrease in them, together with the increase of the imposed potential in the anodic oxidation process.

If the electrochemically polished Al1050 aluminum alloy surface has a determined wear rate of 0.955 mm^3^, a wear volume of only 0.053 mm^3^ has been determined for the nanoporous aluminum oxide film obtained at 15 V imposed potential. The increase of potential from 15 V to 18 V or 21 V during the anodic oxidation process results in a decrease of wear volume nanoporous aluminum oxide films to 0.017 mm^3^ and 0.015 mm^3^ respectively.

According to Fig. 8a the wear volumes maintain a decreasing trend of the values simultaneously with the increase of the imposed potential in the anodic oxidation process. The amount of wear volume loss by the nanoporous aluminum oxide film obtained at the imposed potential of 15 V show a value of 0.052 mm^3^, much lower as compared to the amount of wear volume loss registered by the electrochemically polished aluminum alloy which value is 0.955 mm^3^. The increase in the imposed potential during anodic oxidation process from 15 V to 18 V or 21 V causes the wear volumes losses to be reduced to 0.017 mm^3^ and 0.015 mm^3^ respectively.

The analysis of the graphs shown in Fig. 8a and b shows a decreasing trend of the amount of wear as well as of wear rate with the increase of the potential value imposed in the anodic oxidation process. Electrochemically polished Al1050 aluminum substrate exhibited a wear rate equal to 6.364 × 10^−3^ mm^3^ Nm^−1^. Nanoporous aluminum oxide film obtained at imposed potentials of 15 V, 18 V and 21 V exhibited lower wear rates from 0.349 × 10^−3^ mm^3^ Nm^−1^, to 0.112 × 10^−3^ mm^3^ Nm^−1^ and respectively to 0.099 × 10^−3^ mm^3^ Nm^−1^.

## Conclusions

The evaluation of the mechanical properties of the nanoporous aluminum oxide films compared to the mechanical properties of the electrochemically polished Al1050 alloy substrate and the influence of the imposed potentials for obtaining the nanoporous aluminum oxide films on the friction and wear resistance was carried out in the present paper.

From the analysis of the friction coefficient diagrams resulted from sliding of corundum ball on tested surfaces in dry condition, nanoporous aluminum oxide films have been found to exhibit lower friction coefficients as compared to the electrochemically polished Al1050 alloy substrate. Also, an increase in the imposed potential during anodic oxidation process results in decreased friction coefficient values.

The SEM micrographs reveal a decrease in the average values of the wear track width formed on the surfaces of the nanoporous aluminum oxide films as compared to the average value of the wear track width formed on the surface of the electrochemically polished Al1050 alloy substrate. Increasing the imposed potential during the anodic oxidation process results in decreased average values of the wear track diameters.

From the analysis of SEM images it is noted that the electrochemically polished Al1050 alloy substrate exhibits an adhesive wear mechanism, while the nanoporous aluminum oxide films obtained at 15 V potential both abrasive and an adhesive wear mechanism exhibits. The nanoporous oxide film obtained at 18 V and 21 V show only an abrasive wear mechanism.

Increasing the imposed potential during anodic oxidation process results in the formation of more uniform and more compact tribo-layers on the surfaces of the nanoporous aluminum oxide films.

## Conflicts of interest

There are no conflicts to declare.

## Supplementary Material
